# LAMC1 aggravates diabetic retinopathy through PI3K/AKT signaling-regulated epithelial-mesenchymal transition in retinal pigment epithelial cells

**DOI:** 10.1016/j.jphyss.2025.100045

**Published:** 2025-10-01

**Authors:** Lei Liu, Yanlin Gao, Shiqi Yao

**Affiliations:** a Tianjin Eye Hospital, Tianjin 300020, PR China; b Tianjin Key Lab of Ophthalmology and Visual Science, Tianjin 300020, PR China

**Keywords:** Diabetic retinopathy, PI3K/Akt signaling pathway, EMT, LAMC1

## Abstract

Diabetic retinopathy (DR), a leading cause of adult blindness, with LAMC1-mediated epithelial-mesenchymal transition (EMT) playing a key role. By analyzing DR-related microarray datasets (GSE60436/GSE102485) from GEO, we identified 685 differentially expressed genes (570 downregulated, 115 upregulated). Functional and WGCNA analyses linked these to PI3K/Akt signaling, revealing 11 diagnostic hub genes, including LAMC1. Western blot analysis confirmed that LAMC1 significantly upregulated in high glucose (HG)-treated ARPE-19 cells and diabetic mouse retinas. In vitro and in vivo experiments confirmed that LAMC1 promotes EMT in retinal pigment epithelial (RPE) cells via PI3K/Akt activation, enhancing migration and invasion. Conversely, LAMC1 knockdown alleviated retinal damage in diabetic mice. Our studies uncovered that LAMC1’s role in DR progression through PI3K/Akt-driven EMT, suggesting its potential as a therapeutic target.

## Highlights

• LAMC1 promotes diabetic retinopathy through epithelial-mesenchymal transition regulated by PI3K/AKT signaling axis.

## Graphical Abstract

**Figure:**
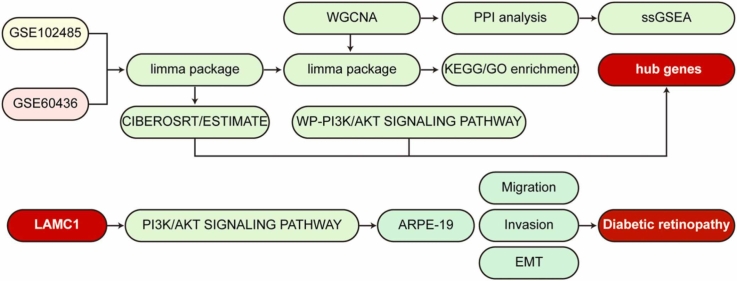

## Introduction

Diabetes mellitus is a chronic metabolic disorder defined by persistent hyperglycemia, primarily stemming from pancreatic β-cell dysfunction leading to inadequate insulin biosynthesis or secretion [1]. Its complications exert detrimental effects on various bodily organs, including the liver, heart, kidneys, joints, and others [2], [3], [4], and also extend to the eyes [5]. Diabetic retinopathy (DR) is a prevalent diabetes-related complication and a significant contributor to visual impairment [6]. In recent decades, the global incidence of diabetes has surged, with a diabetic population of 366 million in 2011, a number projected to surpass 500 million by 2025 [7]. Consequently, an escalating number of individuals are at risk of encountering diabetic microvascular complications. The progression of DR spans from initial vascular abnormalities to the proliferative stage, culminating in vision impairment and, in severe cases, blindness [8]. Presently, the primary strategy for preventing DR centers on meticulous glycemic control. For patients diagnosed with DR, laser photocoagulation represents a treatment option to arrest disease progression; however, this approach carries potential drawbacks, including constriction of the visual field, degradation of color vision, and moderate reductions in visual acuity [9]. Hence, the imperative lies in the early and precise diagnosis and treatment of DR. Furthermore, recent insights into the disease highlight the pressing need for preventive interventions aimed at delaying the progression of early-stage disease.

The epithelial-mesenchymal transition (EMT) serves as a central mechanistic driver in the pathogenesis of ocular fibrotic disorders, with demonstrated involvement in diabetic retinopathy-associated neovascularization and lens epithelial cell transdifferentiation during cataract formation [10]. The retinal pigment epithelium (RPE), a polarized epithelial monolayer situated at the retinal-choroidal interface, maintains mitotic quiescence under homeostatic conditions while orchestrating photoreceptor support and immune regulation. Nevertheless, upon exposure to physical or pathological stimuli, RPE cells are capable of proliferating rapidly and undergoing EMT. This EMT process is marked by the disappearance of epithelial characteristics and the attainment of mesenchymal phenotypes [11]. Emerging evidence highlights the upregulation of EMT-associated signaling pathways and protein markers in PDR [12]. For example, experimental investigations that employ high - glucose settings to simulate the diabetic microenvironment have shown a substantial increase in the expression of mesenchymal markers. In cultured RPE cells, markers like α-smooth muscle actin (α-SMA) and vimentin are notably upregulated [13]. These findings underscore the dynamic involvement of EMT in RPE dysfunction and retinal fibrotic changes during DR advancement.

The PI3K/AKT signaling cascades has been mechanistically implicated in modulating EMT of retinal RPE cells during DR pathogenesis, as evidenced by multiple experimental validations [14], [15]. Through integrated bioinformatics analysis of DR transcriptomic datasets, we systematically identified 11 core regulatory genes within the PI3K/AKT signaling axis. Subsequent qPCR validation in high glucose-treated ARPE-19 cells confirmed differential expression patterns of these candidate genes, with laminin subunit gamma 1 (LAMC1) demonstrating the most pronounced upregulation under hyperglycemic conditions. LAMC1 encodes the gamma-chain constituent of laminin isoforms, previously associated with diabetic nephropathy progression through its extracellular matrix remodeling functions [16]. Emerging evidence delineates LAMC1's pleiotropic roles in potentiating angiogenesis via Wnt/β-catenin-mediated endothelial cell migration [17], and driving EMT processes in epithelial malignancies including triple-negative breast carcinoma and cutaneous squamous cell carcinoma [18], [19]. However, the underlying mechanism that How LAMC1 regulates EMT of RPE cells through PI3K/AKT in DR remains elusive.

Collectively, we postulate that LAMC1 exacerbates DR progression by driving PI3K/AKT-mediated EMT in RPE cells.

## Materials and methods

### Data source

The gene expression data relevant to patients with DR were obtained from the Gene Expression Omnibus (GEO) repository. This repository is maintained by the National Center for Biotechnology Information (NCBI) and can be accessed at the following link: https://www.ncbi.nlm.nih.gov/geo/. We specifically chose microarray gene expression profiles from GSE60436 and GSE102485 [20], [21]. Probe platform files were acquired, and probes corresponding to the same gene were subjected to averaging. After preprocessing, the GSE60436 dataset consisted of 6 samples from patients with DR and 3 normal control samples. In contrast, the GSE102485 dataset comprised 22 DR samples and 3 normal control samples. Furthermore, genes related to the PI3K/Akt signaling pathway were obtained from the Gene Set Enrichment Analysis (GSEA) platform, which can be accessed at the website http://www.gsea-msigdb.org/gsea/index.jsp.

### Data integration and differential expression analysis

Before merging datasets, we standardized each batch of data, then we utilized the "remove Batch Effect" function in the R software to eliminate batch effects inherent in the GSE60436 and GSE102485 datasets. Subsequently, data normalization was executed through the "normalize Between Arrays" function. Following these steps, gene name annotation was carried out, and genes that were either duplicated or devoid of expression values were excluded from the processed dataset. In the GSE3307 dataset, a differential gene expression analysis was carried out to contrast samples from DR patients with normal samples. The "limma" package was employed for this analysis, with the criteria of log2|(FC)|≥ 1 and an adjusted p-value less than 0.05.

### Gene functional enrichment analysis

Functional annotation, which included the analysis of Gene Ontology (GO) terms and Kyoto Encyclopedia of Genes and Genomes (KEGG) pathways, was executed using the R package known as "clusterProfiler". Terms and pathways that showed enrichment were considered statistically significant when they met the following criteria: the minimum size of the gene set was 5, the maximum size of the gene set was 5000, the p-value was less than 0.05, and the false discovery rate (FDR) was less than 0.25.

### Weighted gene co-expression network analysis (WGCNA)

WGCNA was carried out utilizing the R package "WGCNA" to establish a co-expression network. The process encompassed sample clustering, the removal of outliers, and the determination of the soft thresholding parameter to establish scale-free network relationships. The topological overlap matrix (TOM) was utilized in place of the adjacency matrix in order to reduce spurious correlations. Genes were subjected to hierarchical clustering according to dissimilarity metrics. To obtain meaningful modules, a minimum module size of 30 was established during this clustering process. Module Eigengenes (MEs) were computed and consolidated with a height cutoff of 0.75 to identify closely correlated modules. The module that had the closest association with DR was identified through the evaluation of the correlations between the modules and the clinical features. This identification process adhered to a specific threshold: a correlation coefficient greater than 0.5 and a p-value less than 0.05. Lastly, the individual gene module membership (MM) and gene significance (GS) were measured. Genes that satisfied the criteria of an absolute value of MM greater than 0.8 and an absolute value of GS greater than 0.2 within the crucial modules were chosen for further analysis.

### Protein–protein interaction network analysis (PPI)

An online tool named Metascape (https://metascape.org/gp/index.html#/main/step1) was utilized to construct the protein - protein interaction (PPI) network for the module genes. Subsequently, the Molecular Complex Detection (MCODE) algorithm was applied to identify the hub genes within this network.

### Single-sample gene set enrichment analysis (ssGSEA)

The R package "GSVA" was harnessed to compute scores for each sample via the ssGSEA algorithm. Subsequently, comparisons were made to gauge score variations across samples.

### Immune infiltration scoring

Leveraging our expression profiles, we employed the R packages CIBERSORT and ESTIMATE to compute immune infiltration scores for each sample.

### Cell culture

Human retinal pigment epithelial ARPE-19 cells were sourced from the China Center for Type Culture Collection (CCTCC, located in Shanghai, China). These cells were cultured in a 1:1 mixture of DMEM/F12 medium (manufactured by Gibco, USA) supplemented with 10 % FBS (from Gibco) and 1 % penicillin/streptomycin (from Gibco). The cell culture was maintained in a humidified incubator set at a temperature of 37°C and with a 5 % CO₂ atmosphere. In accordance with the experimental design, the cells were incubated under two different glucose conditions: one group was exposed to 5.5 mM glucose, designated as the normal glucose (NG) group, while the other group was incubated with 30 mM glucose, referred to as the high-glucose (HG) group.

### Cell transfection

The sh-NC and sh-LAMC1 plasmids were designed and chemically synthesized by GenePharma, a company based in Shanghai, China. pTSB-U6-shRNA-EF1-copGFP-2A-PURO-TSB204084–1 is the plasmid vector, U6 is the promoter, and the sequence of sh-LAMC1 is GCGCCTATAACTTTGACAATA. ARPE-19 cells were initially seeded in 6-well plates and then placed in a cell incubator for a 24-hour cultivation period. Subsequently, these cells were transfected with the aforementioned plasmids using Lipofectamine® 3000 reagent (produced by Invitrogen, USA). The transfection procedure was carried out in strict accordance with the manufacturer's provided protocols. 48 h after the transfection, the cells were collected. This collection was aimed at evaluating the transfection efficiency and conducting subsequent experimental analyses.

### Animals model construction

Male C57BL/6 J mice at 8 weeks of age were procured from the Shanghai Laboratory Animal Research Center in Shanghai, China. These mices were then accommodated within a specific-pathogen-free (SPF) facility, where they had unrestricted access to food and water. The environmental conditions within this facility were strictly regulated: the temperature was maintained at 22 ± 2°C, the relative humidity ranged from 50 % to 60 %, and a 12-hour light/dark cycle was implemented. All animal-related experimental procedures were approved by the Animal Ethics Committee of Tianjin Eye Hospital (2025-SYDWLL-000401). Following a 7-day period of adaptive feeding, the mice were randomly allocated into 4 distinct groups, each consisting of 6 mice per group. These groups were designated as Control, DM, DM+sh-NC, and DM+sh-LAMC1. In the experimental groups, diabetes mellitus (DM) was induced by means of intraperitoneal injection of streptozotocin (STZ). The STZ was dissolved in 10 mM citrate buffer with a pH of 4.5 at a concentration of 50 mg/kg and sourced from Sigma-Aldrich (USA). This injection was administered for 5 consecutive days. In contrast, the Control group mice were injected with an equivalent volume of citrate buffer having a pH of 4.5. Seven days post-STZ, fasting blood glucose (FBG) was measured using an AC6000 analyzer (Audiocom Medical, Jiangsu, China), and DM was confirmed at FBG ≥ 300 mg/dL. Two weeks after DM confirmation, intravitreal injection of 2 μL AAV2 vectors (2 ×10⁹ vg, GenePharma) containing sh-LAMC1 or sh-NC was delivered using a 33-gauge disposable needle under isoflurane anesthesia. Six weeks subsequent to the streptozotocin injection, which corresponded to 8 weeks after the diagnosis of diabetes mellitus, the mice were humanely euthanized. At this time, retinal tissues were harvested. One part of the retinal tissues was fixed in 4 % paraformaldehyde solution, which was intended for hematoxylin and eosin (H&E) staining. The other part was rapidly frozen at - 80°C and was reserved for Western blot analysis.

### Western blot

The ARPE-19 cells and retinal tissues were subjected to lysis using RIPA lysis buffer (produced by Beyotime, China) while being maintained on ice. Following the lysis process, the samples were centrifuged, and subsequently, the protein concentration of the supernatants was quantified using the BCA kit (also from Beyotime). Subsequently, protein samples of equal quantity were separated via SDS - PAGE. After that, the separated proteins were transferred onto PVDF membranes (supplied by Merck Millipore, USA). The PVDF membranes were first blocked by incubating them with 5 % milk (from Beyotime, China). Then, they were incubated overnight at 4 °C with specific primary antibodies as listed in Table 1. Following this, the membranes were washed with Tris buffered saline with TBST buffer. Finally, they were allowed to react with the secondary antibody for 2 h at room temperature. After that, chemiluminescent (ECL) reagent (Thermo Scientific, USA) was used to expose the membranes. The images were captured by ChemiDoc MP system (Bio-rad, USA) and analyzed using ImageJ software (National Institutes of Health, USA).

**Table 1:** Primary antibodies used in Western blotting assay.

**Antibody**	**Company (Cat. No.)**	**Working dilution**
LAMC1	Cell Signaling Technology (92921)	1:1000
E-cadherin	ABclonal (A20798)	1:1000
N-cadherin	Proteintech (22018–1-AP)	1:2000
Vimentin	Abcam (ab92547)	1:1000
Snail	ABclonal (A11794)	1:1000
PI3K	Cell Signaling Technology (4292)	1:1000
p-PI3K	Affinity Biosciences (AF3242)	1:1000
AKT	Cell Signaling Technology (9272)	1:1000
p-AKT	Cell Signaling Technology (9271)	1:1000
GAPDH	ABclonal (A19056)	1:10000
Goat Anti-Rabbit IgG H&L (HRP)	ABclonal (AS014)	1:10000

### Transwell assay

The invasion assay was carried out using Transwell inserts with an 8 - μm pore size in 24 - well plates, which were sourced from Corning (USA). In brief, the ARPE-19 cells were first transfected with either sh-NC or sh-LAMC1 plasmids. After a 72-hour transfection period, the cells were seeded into the upper chambers of a Transwell plate that had been coated with Matrigel (BD Biosciences, USA). The seeding density was set at 3 × 10⁵ cells per well, and the cells were placed in a serum-free medium. The lower chambers were filled with DMEM/F12 (1:1) medium (Gibco) containing 10 % FBS. In the HG group, 30 mM glucose was added to the medium, and the control group was treated with 5.5 mM glucose. Following a 72-hour incubation period in an incubator maintained at 37°C with a 5 % CO₂ atmosphere, the non-migrated cells as well as the residual Matrigel present on the upper surface of the upper chambers of the Transwell inserts were carefully removed using a cotton swab. Subsequently, the chambers were rinsed three times with PBS. The cells that had migrated to the lower surface of the lower chambers were then fixed with 4 % paraformaldehyde for a duration of 30 min. After fixation, the cells were stained with 0.2 % crystal violet for 20 min. The relative cell migrations were then observed and quantified under a light microscope.

### Wound healing assay

The ARPE-19 cells transfected with sh-NC or sh-LAMC1 plasmids for 72 h were seeded in six-plate at a density of 1 × 10^5^ cells/mL. When confluence reaches above 90 %, scratch a wound via a 100 μL pipette tip. Subsequently, washing the cells with PBS and the cells was subjected to different interventions. At the time points of 0 h and 72 h, wound images were taken with the aid of a low-magnification phase-contrast microscope manufactured by Olympus, Japan. In order to evaluate the cell migration, the process of wound closure was quantitatively analyzed. This analysis was carried out by utilizing ImageJ software, which is provided by the National Institutes of Health.

### Hematoxylin-eosin (H&E) staining

Th retinal tissues were initially fixed in a 4 % paraformaldehyde solution. Subsequently, they underwent a dehydration process using gradient ethanol. After dehydration, the tissues were cleared with xylene and then embedded in paraffin. Once embedded, the paraffin blocks were sectioned into multiple 4-micrometer-thick paraffin slices. These slices were then baked at 60°C to ensure proper adhesion. Following baking, the slices were dewaxed to remove the paraffin and then stained using an HE staining kit from Solarbio (China). Finally, images of the stained slices were captured by a microscope from Olympus Corporation in Japan. For each section, 5 central fields were selected and imaged to obtain representative views of the tissue structure.

### Immunofluorescence

Retinal tissue samples from male C57BL/6 mice were fixed overnight in 4 % paraformaldehyde at 4°C, followed by sequential dehydration in an ethanol gradient, xylene clearing, paraffin embedding, and sectioning at 4 μm thickness. Following fixation, tissue sections were permeabilized with 0.1 % Triton X-100 (Beyotime) for 30 min and subsequently blocked using 1 % BSA (Beyotime) at room temperature for 1 h. Then, the tissue sections were respectively incubated with anti-LAMC1antibody (PA5–101334, 1:300, thermofisher), Rabbit anti-E-cadherin (ab308347, 1:500, abcam) and Rabbit anti-N-cadherin (22018–1-AP, 1:200, Proteintech) in block buffer for overnight at 4°C. Tissue sections were incubated with Goat Anti-Rabbit IgG H&L Alexa Fluor® 555 (ab150078, 1:500, Abcam) and Alexa Fluor® 488 (ab150077, 1:1000, Abcam) for 2 h at room temperature. Following two PBS washes, nuclei were counterstained with DAPI.

### Statistical analysis

All statistical analyses in this study were executed using the R software. The Mann-Whitney test was applied for intergroup gene expression comparisons. The "pROC" package facilitated the construction of diagnostic models and the generation of ROC curves. Unless otherwise specified, a significance level of *P* <0.05 was established as the threshold for statistical significance. All of the experimental findings were analyzed by means of GraphPad Prism software (USA). The results were presented in the form of mean ± standard deviation (SD). For the comparison between two groups, a two-tailed paired Student's *t*-test was employed. A p-value< 0.05 was considered statistically significant (*P < 0.05, **P value < 0.01, ***P < 0.001).

## Results

### Data integration and analysis of differential expression

**Fig. 1:**
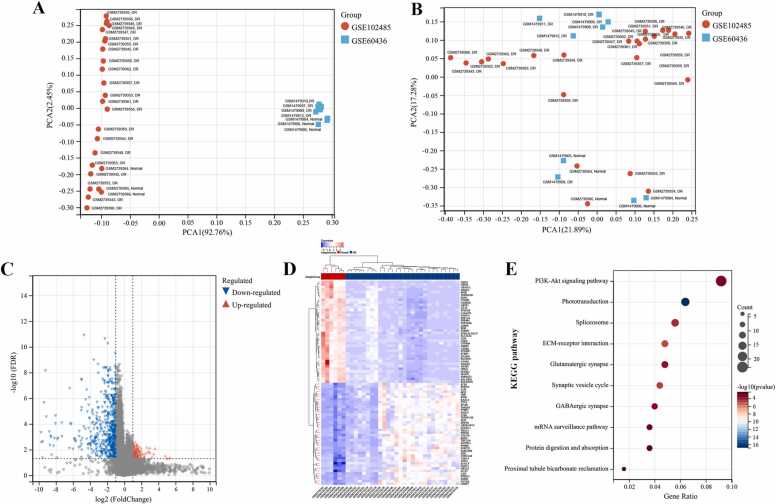
Data Integration and Differential Expression Analysis. A: PCA analysis before dataset merging; B: PCA analysis after dataset merging; C: Volcano plot depicting differentially expressed genes; D: Heatmap illustrating differentially expressed genes. E: KEGG pathway enrichment analysis of differentially expressed genes.

Normalization of expression values in the GSE60436 and GSE102485 datasets was achieved through Principal Component Analysis (PCA). Visual representations of data before and after normalization are depicted in Fig 1A and B, respectively. Subsequently, a differential expression analysis was performed on the merged dataset, and the outcomes are visualized in Fig. 1C, using a threshold of |log2 FC= ≥ 1 and adjusted p-value < 0.05. In total, 685 genes exhibited differential expression, comprising 570 downregulated and 115 upregulated genes. The heatmap in Fig. 1D illustrates the top 50 differentially expressed genes.

### Functional enrichment analysis of differentially expressed genes

In order to clarify the functions related to these genes that exhibited differential expression, KEGG enrichment analyses were carried out. The KEGG analysis demonstrated that the differentially expressed genes were mainly concentrated in several pathways. These included the PI3K/Akt signaling pathway, which plays a crucial role in various cellular processes such as cell growth, proliferation, and survival. Additionally, the genes were enriched in the phototransduction pathway, which is essential for the conversion of light into electrical signals in the retina. Moreover, the spliceosome pathway, which is involved in the processing of pre-mRNA to mature mRNA, also showed significant enrichment of these differentially expressed genes. (Fig. 1E).

### Construction of a gene co-expression network for DR patients using WGCNA

**Fig. 2:**
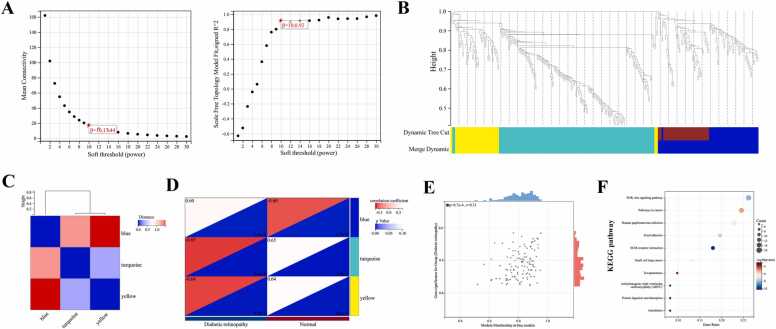
Construction of a Gene Co-Expression Network for DR Patients Using WGCNA. A: Scale independence and mean connectivity; B: Gene dendrogram and module identification before and after merging; C: Module eigengene clustering; D: Correlation between modules and clinical phenotypes; E: Scatter plot of MM and GS for the Blue module. F: KEGG pathway enrichment analysis of module genes.

Utilizing the R package WGCNA, a co-expression network analysis was conducted on the dataset. The process involved hierarchical clustering of all samples, removal of outlier samples, and selection of a soft threshold value of β= 10 to establish a scale-free network (Fig. 2A). The generation of a gene dendrogram based on topological overlap is presented in Fig. 2B, leading to the identification of three modules (Fig. 2C). Subsequently, by assessing the Pearson correlation heatmap between modules and clinical phenotypes (DR), it was determined that the Blue module exhibited the highest correlation (cor=0.60, p = 1.9e-4) (Fig. 2D). Furthermore, an analysis of genes within the Blue module and their correlation with DR clinical phenotypes revealed a significant association (cor=0.30, p = 8.7e-4) (Fig. 2E). Consequently, the MEskyblue module was regarded as relevant to DR. A total of 65 genes from this module met the criteria of |MM|> 0.8 and |GS| > 0.2 and were designated as module genes for further analysis. Subsequently, an exploration of functional and molecular pathway enrichment was conducted on the 65 selected genes within the Blue module. KEGG analysis indicated that these genes were primarily enriched in pathways including the PI3K/Akt signaling, pathways in cancer, and human papillomavirus infection (Fig. 2F). Additionally, GO enrichment analysis uncovered significant enrichment in functions such as signaling receptor binding, extracellular region, and immune system processes (Figure S1A, S1B, S1C).

### Identification of hub genes

**Fig. 3:**
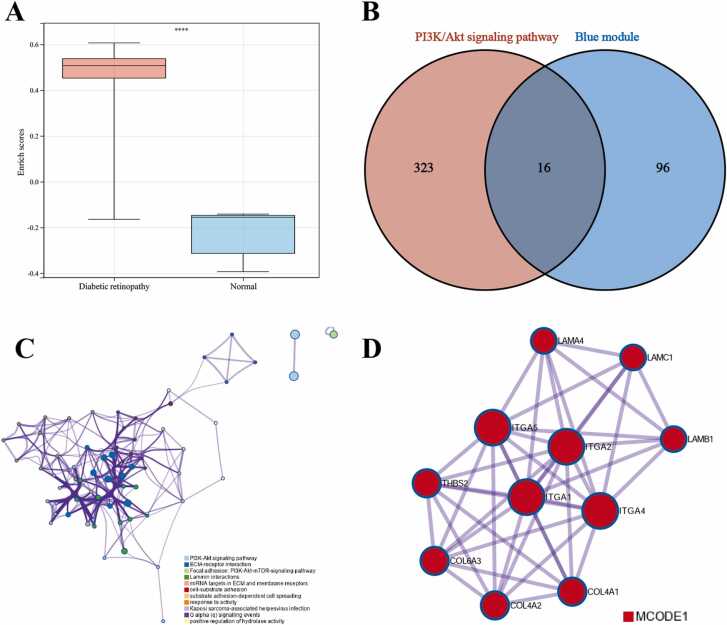
Identification of Hub Genes. A: ssGSEA scores for PI3K/Akt signaling pathway-related genes; B: Intersection genes between PI3K/Akt signaling pathway-related genes and Blue module genes; C: PPI network constructed from intersectiongenes; D: Significant module identified using the MCODE method. ****P < 0.0001.

The relevant genes for the PI3K/Akt signaling pathway were retrieved from the GSEA database under WP_PI3KAKT_SIGNALING_PATHWAY. Subsequently, ssGSEA analysis was employed to calculate pathway-related scores for both DR patients and the control group, revealing significantly higher scores among DR patients (Fig. 3A). Subsequently, 16 genes common to both the PI3K/Akt signaling pathway-related genes and the genes within the Blue module were identified (Fig. 3B). These intersecting genes were employed to construct a PPI network, with significant modules identified using the MCODE algorithm (Fig 3C and D). The MCODE1 module included 11 genes, specifically COL4A1, COL4A2, COL6A3, ITGA1, ITGA2, ITGA4, ITGA5, LAMA4, LAMB1, LAMC1, and THBS2. This extracted MCODE1 component was notably associated with ECM-receptor interaction, PID INTEGRIN1 PATHWAY, and focal adhesion (Table 2).

**Table 2:** Pathway and process enrichment analysis in MCODE component.

MCODE	GO	Description	Log10(P)
MCODE1	hsa04512	ECM-receptor interaction	−28.2
MCODE1	M18	PID INTEGRIN1 PATHWAY	−25.9
MCODE1	hsa04510	Focal adhesion	−24.0

### Expression of hub genes and their diagnostic value

**Fig. 4:**
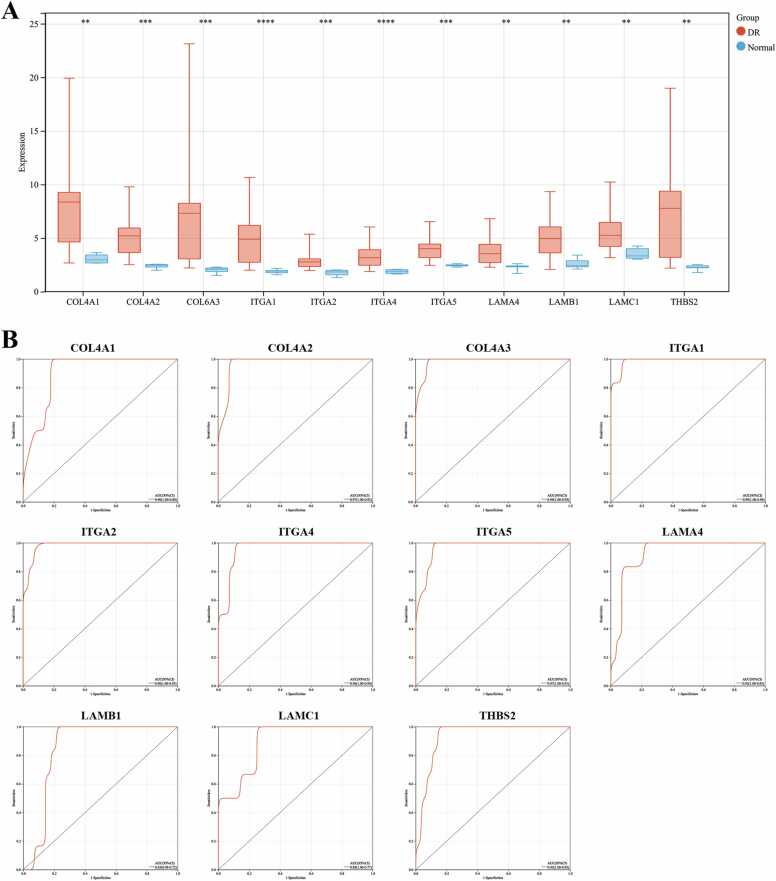
Expression of Hub Genes and Diagnostic Value. A: Expression levels of hub genes in DR; B: ROC curves for hub genes in diagnosing DR. **P < 0.01, ***P < 0.001, ****P < 0.0001.

The expression levels of COL4A1, COL4A2, COL6A3, ITGA1, ITGA2, ITGA4, ITGA5, LAMA4, LAMB1, LAMC1, and THBS2 were compared between DR samples and normal control samples within the dataset. In the given dataset, each of the 11 genes demonstrated remarkably elevated expression levels when analyzed in the DR samples as opposed to the normal control samples (Fig. 4A). Subsequently, to evaluate the diagnostic potential of these 11 hub genes, the area under the curve (AUC) values were calculated. All 11 hub genes demonstrated AUC values exceeding 0.85, affirming their high diagnostic efficacy for DR (Fig. 4B).

### Assessment of immune infiltration levels in DR

The CIBERSORT method was applied to determine immune cell infiltration scores for 22 distinct immune cell types in both DR patients and healthy controls. The outcomes revealed noteworthy disparities in the infiltration levels of Plasma cells, Monocytes, and Macrophages M2 between DR patients and the normal group (Figure S2A). Additionally, the ESTIMATE algorithm facilitated the computation of immune and stromal scores for both DR patients and the normal group, illustrating significantly elevated immune and stromal scores in DR patients (Figure S2B).

### Correlation between hub genes and immune scores

Based on the median expression levels of the 11 hub genes in DR samples, DR patients were categorized into high-expression and low-expression groups. Subsequent examination indicated significant differences in stromal scores between the high and low-expression groups of these 11 genes, while no significant distinction was observed in immune scores (Figure S3).

### Hub genes were validated in HG stimulated ARPE-19 cell lines

**Fig. 5:**
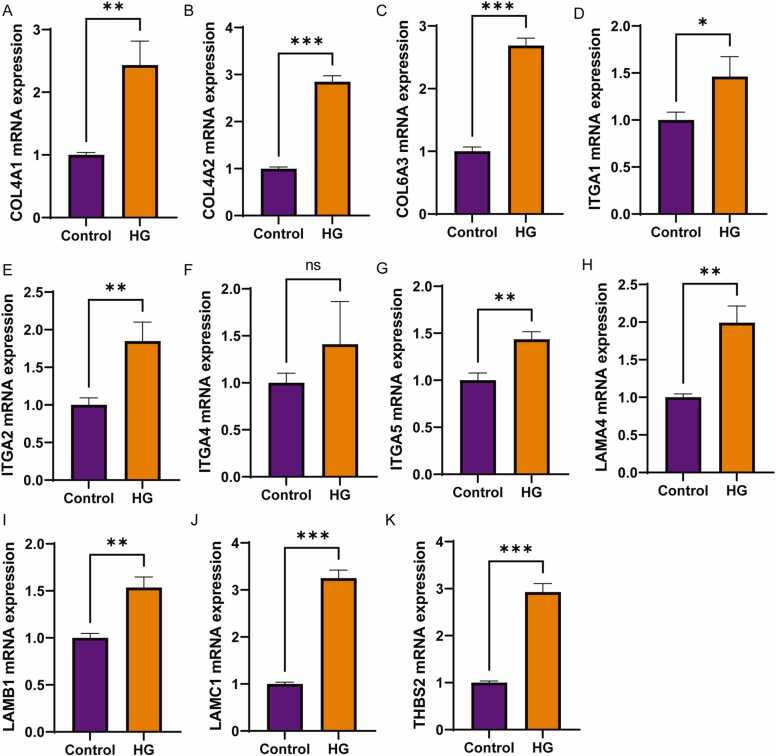
qPCR validation of 11 hub genes in HG stimulated ARPE-19 cell line. A-K: qPCR detected the mRNA levels of COL4A1, COL4A2, COL6A3, ITGA1, ITGA2, ITGA4, ITGA5, LAMA4, LAMB1, LAMC1, and THBS2 in ARPE-19 cells. P-value were calculated using two-tailed Student’s *t*-test. Data were displayed as mean ± SD. *p < 0.05, **p < 0.01, ***p < 0.001.

ARPE-19 cells were exposed to HG environment to establish an *in vitro* model that emulates DR. qPCR analysis of the 11 candidate hub genes demonstrated significant upregulation of 10 genes under high glucose (HG) conditions compared to controls. Obviously, HG treatment increased ITGA4 expression, but no statistically significant difference was observed compared to the control group (Fig. 5F). These findings demonstrate that while the majority of hub genes exhibit responsiveness to hyperglycemic conditions in this in vitro diabetic retinopathy model, ITGA4 may function through a distinct regulatory mechanism or display reduced susceptibility to direct glucose-mediated regulation under these experimental parameters. Among the others, LAMC1 emerges as a critical focus for DR research due to its unique role in retinal basement membrane (BM) stability, pathological angiogenesis, and neurovascular injury [22], [23], [24]. Notably, LAMC1 exhibits upregulated expression in diabetic nephropathy and has been demonstrated to regulate EMT in cancer progression [16], [18], [19]. Given the critical role of EMT in RPE dysfunction during DR, these findings collectively suggest a potential mechanistic link between LAMC1 overexpression and EMT-driven pathological alterations in DR.

### Knockdown of LAMC1 inhibits high glucose-induced EMT in ARPE-19 cells

**Fig. 6:**
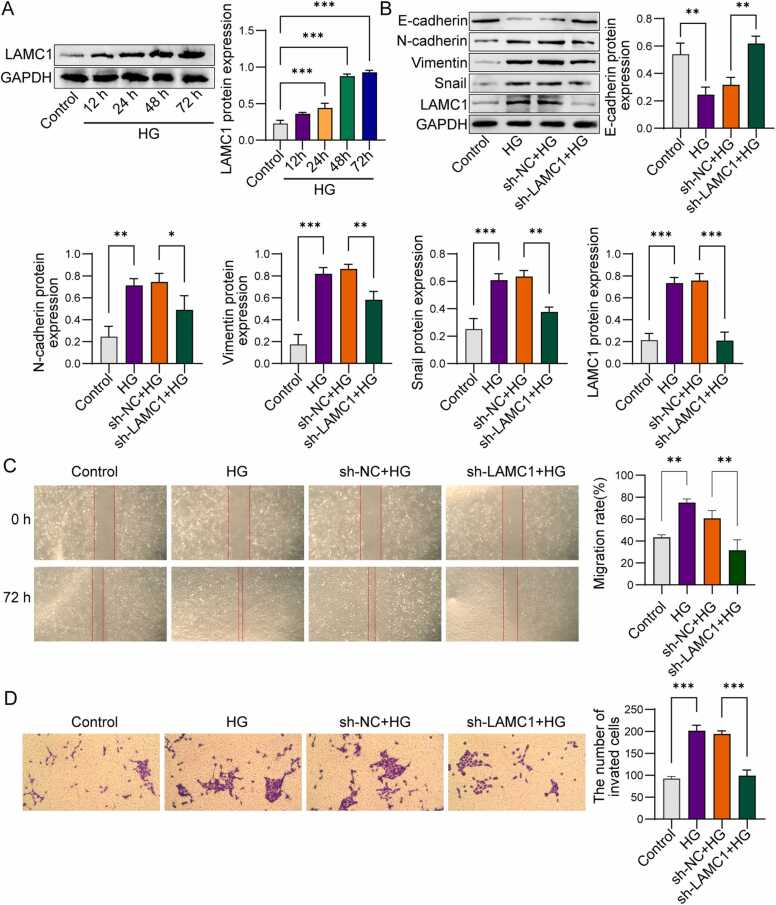
Knockdown of LAMC1 inhibits high glucose-induced EMT in ARPE-19 cells. A: Western blot analysis of LAMC1 expression in ARPE-19 cells. B: Western blot analysis of LAMC1 and EMT-related proteins (E-cadherin, N-cadherin, Vimentin, and Snail) in ARPE-19 cells. C: Scratch assay to evaluate cell migration ability. D: Transwell assay to assess cell invasion capability. The images show the invasive cells under different treatment conditions. Experiments were repeated three times independently (n = 3). P-value were calculated using two-tailed Student’s *t*-test. Data were displayed as mean ± SD. *p < 0.05, **p < 0.01, ***p < 0.001.

Further investigation is warranted to elucidate whether LAMC1 directly modulates EMT in RPE cells under hyperglycemic conditions, which may unveil novel therapeutic targets for mitigating DR progression. Strikingly, LAMC1 expression gradually increased with prolonged HG treatment compared to the control group (Fig. 6A). Subsequently, the effect of LAMC1 on EMT in ARPE-19 cells under HG conditions was investigated. In contrast to the control group, the administration of HG led to a notable decline in the expression of E-cadherin. Simultaneously, it caused an increase in the levels of N-cadherin, Vimentin, and Snail. These changes collectively contributed to the promotion of EMT (Fig. 6B). In addition, knockdown of LAMC1 reduced the proportion of cell migration and cell invasion induced by HG (Fig 6C and D). These results indicate that knockdown of LAMC1 inhibits high glucose-induced EMT in human RPE cells.

### LAMC1 promotes high glucose-induced EMT in ARPE-19 cells through the PI3K/AKT pathway

**Fig. 7:**
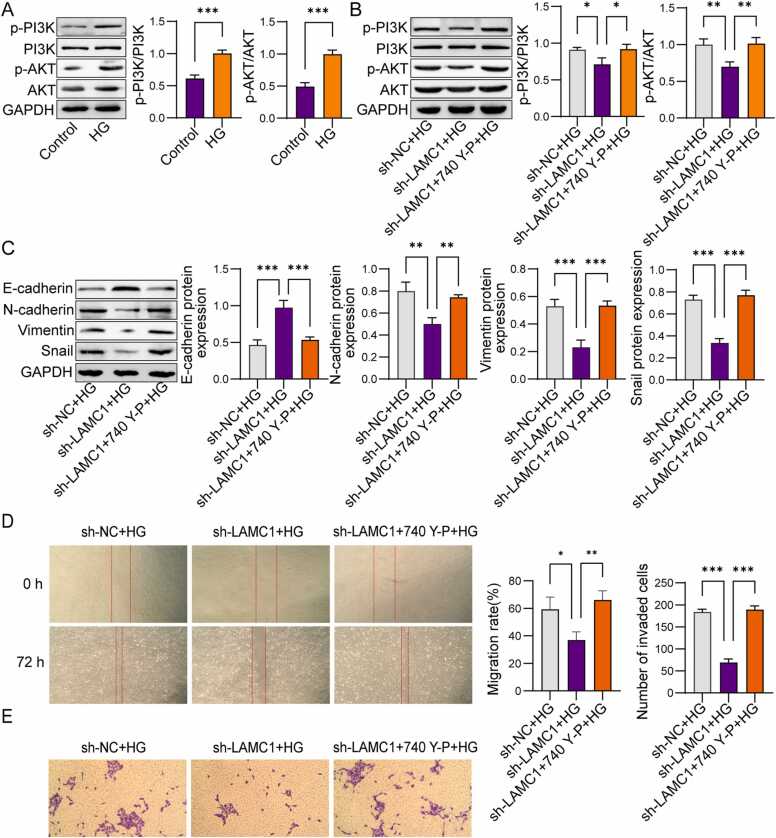
LAMC1 promotes high glucose-induced EMT in ARPE-19 cells through the PI3K/AKT pathway. A-B: Western blot analysis of p-PI3K and p-AKT levels in ARPE-19 cells. C: Western blot analysis of EMT-related proteins in ARPE-19 cells. D: Scratch assay to evaluate cell migration ability. E: Transwell assay to assess cell invasion capability. Experiments were repeated three times independently (n = 3). P-value were calculated using two-tailed Student’s *t*-test. Data were displayed as mean ± SD. *p < 0.05, **p < 0.01, ***p < 0.001.

Furthermore, it was found that the PI3K/AKT signaling pathway was activated by the action of high glucose (HG) in ARPE-19 cells (Fig. 7A). Thus, the relationship between LAMC1 and the PI3K/AKT signaling pathway was investigated. Compared to the sh-NC+HG group, the sh-LAMC1 +HG group showed significantly reduced expression of p-PI3K and p-AKT. The addition of the PI3K/AKT activator 740 Y-P reactivated this signaling pathway (Fig. 7B). Therefore, we investigated whether LAMC1 affects the EMT, migration, and invasion of ARPE-19 cells through the PI3K/AKT pathway. Consistent with these findings, knockdown of LAMC1 inhibited cell EMT, as well as cell migration and invasion (Fig. 7C-E). Moreover, knockdown of LAMC1 followed by treatment with 740 Y-P reversed the expression of EMT-related proteins, significantly downregulating E-cadherin and upregulating N-cadherin, Vimentin, and Snail (Fig. 7C), while restoring the HG-induced promotion of cell migration and invasion (Fig 7D and E). These results indicate that LAMC1 promotes high glucose-induced EMT in ARPE-19 cells by activating the PI3K/AKT signaling pathway.

### LAMC1 regulates EMT and the PI3K/AKT pathway in the retina of diabetic mice

**Fig. 8:**
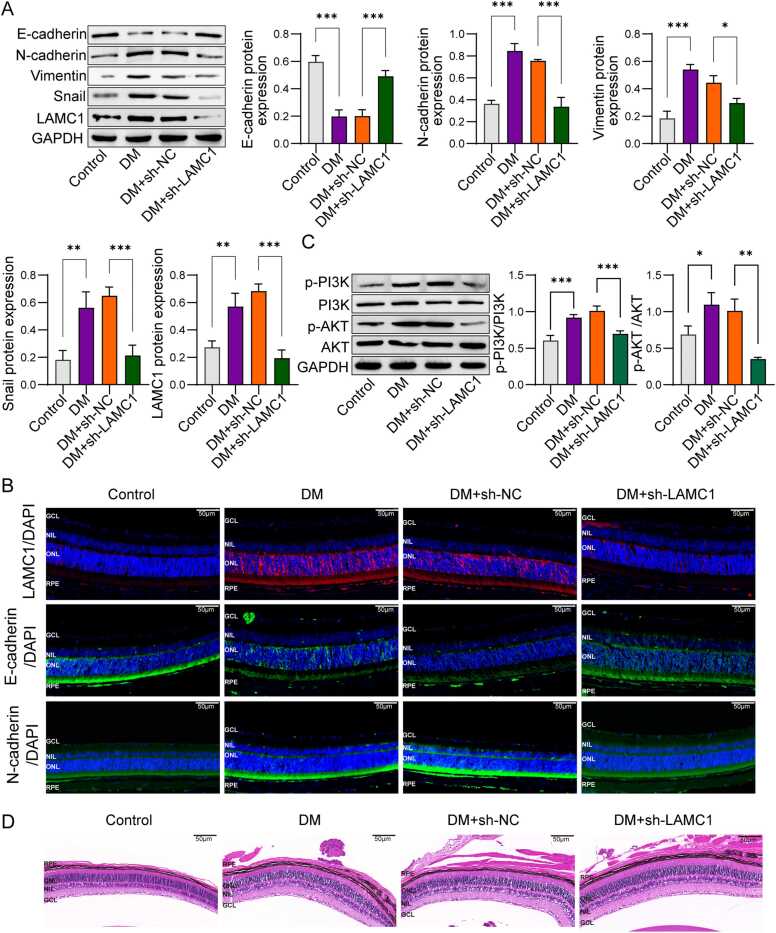
LAMC1 regulates EMT and the PI3K/AKT pathway in the retina of diabetic mice (n = 6). A: Western blot analysis of EMT-related proteins (E-cadherin, N-cadherin, Vimentin, and Snail) in the retina of diabetic mice. B: Immunofluorescence analysis of LAMC1 and EMT markers in retinal tissues. C: Western blot analysis of PI3K/AKT pathway activation in the retina of diabetic mice. D: Hematoxylin and eosin (HE) staining to assess retinal structure in mice. P-value were calculated using two-tailed Student’s *t*-test. Data were displayed as mean ± SD. *p < 0.05, **p < 0.01, ***p < 0.001.

To investigate the *in vivo* regulatory role of LAMC1 in EMT, we established a STZ-induced diabetic murine model, followed by lentiviral-mediated delivery of either control sh-NC or sh-LAMC1. This experimental paradigm enables rigorous assessment of LAMC1-dependent EMT modulation under diabetic conditions with pathophysiological relevance. Consistent with the *in vitro* model, LAMC1 expression was significantly increased in the retinal tissues of DM mice, accompanied by enhanced EMT. Knockdown of LAMC1 inhibited EMT in the retina of DM mice, characterized by upregulation of E-cadherin and significant downregulation of N-cadherin, Vimentin, and Snail (Fig. 8A). To further investigate the role of LAMC1 in epithelial-mesenchymal transition (EMT), we performed immunofluorescence staining for LAMC1, E-cadherin, and N-cadherin in retinal tissues. Consistent with the findings in Fig. 8A, quantitative analysis revealed: (1) significantly enhanced LAMC1 fluorescence intensity in diabetic (DM) mice retinas compared to controls, which was effectively attenuated by sh-LAMC1 treatment; (2) marked downregulation of E-cadherin concomitant with dramatic upregulation of N-cadherin in RPE of DM mice; and complete reversal of these EMT markers following LAMC1 knockdown (Fig. 8B). Moreover, the PI3K/AKT signaling pathway was activated in the retina of DM mice. Knockdown of LAMC1 significantly reduced the expression of PI3K and p-AKT in the retina of DM mice (Fig. 8C). HE staining results showed that, compared to the control group, the inner and outer nuclear layers of the retina in DM mice were disorganized and reduced in number. Knockdown of LAMC1 significantly ameliorated this damage (Fig. 8D). These results indicate that LAMC1 regulates EMT and the PI3K/AKT pathway in the retina of diabetic mice, and knockdown of LAMC1 can alleviate retinal damage in DM mice.

## Discussion

The development and progression of DR entail intricate pathological processes involving abnormalities in cytokines, signaling transduction metabolic enzymes, inflammation, ion channels, and related genes [25]. Increasingly evidences showed that EMT was deeply involved in the formation of fibrous epiretinal membrane in diabetes retinopathy [26]. Thus, elucidating the molecular mechanisms governing EMT regulation is imperative to decipher its precise role in DR pathogenesis and to identify novel therapeutic targets capable of mitigating microvascular dysfunction, fibrotic progression, and pathological angiogenesis. In our study, we identified LAMC1 as a critical regulator of EMT. Moreover, we established that LAMC1 orchestrates EMT progression through precise modulation of the PI3K/AKT signaling axis, revealing a mechanistic link between extracellular matrix components and cellular plasticity.

In our investigation, we initiated the study by identifying 685 differentially expressed genes through the analysis of two GEO datasets. Subsequent functional enrichment analysis of these differentially expressed genes underscored a pronounced enrichment in the PI3K/Akt signaling pathway, accentuating its paramount role in DR progression. Our exploration then delved into the WGCNA of these differentially expressed genes, which led us to identify modules most pertinent to the advancement of DR. Functional enrichment analysis of these module genes reaffirmed their substantial association with the PI3K/Akt signaling pathway, further validating its centrality in DR. These in silico analytical results corroborate earlier research findings in the field. For example, Liu et al. elucidated that the knockdown of PI3K hinders EMT induced by patient-derived vitreous [27]. Similarly, high glucose-induced alterations in EMT and in RPE were rescued in the presence of PI3K and ERK inhibitors [28]. Moreover, our quest eventually pinpointed 11 hub genes through an intersecting analysis of module genes and PI3K-Akt signaling pathway-related genes acquired from the GSEA database. These 11 genes, specifically COL4A1, COL4A2, COL6A3, ITGA1, ITGA2, ITGA5, LAMA4, LAMB1, LAMC1, and THBS2, all exhibited remarkable upregulation in DR and demonstrated a notable capacity for diagnosing the condition. While these genes are prominently expressed in DR, existing evidences indicates that ITGA4, by contrast, is predominantly expressed on immune cells and plays a critical role in inflammatory and immune regulatory processes [29]. Moreover, dysregulation of ITGA4 has been strongly linked to autoimmune diseases such as multiple sclerosis [30] and ulcerative colitis [31], this suggests that it may be differentially expressed in immune cells rather than in retinal epithelial cells in DR. Among the upregulated genes, LAMC1 emerges as a critical focus for DR research due to its unique role in retinal basement membrane (BM) stability, pathological angiogenesis, and neurovascular injury. Research indicates that elevated glucose levels can influence the expression of LAMC1 and alter the biological behavior of retinal choroidal endothelial cells, suggesting LAMC1 may play a critical role in basement membrane alterations associated with diabetic retinopathy [32].

Significantly, it has been reported that LAMC1 is involved in the regulation of EMT in cancer [33], [34]. Thus, we conducted a comprehensive investigation into the regulatory function of LAMC1 in modulating the EMT process, a critical mechanism implicated in the pathogenesis and progression of DR [35]. Obviously, we elucidated for the first time that the silencing of LAMC1 suppresses high glucose-induced EMT in retinal pigment epithelium. Furthermore, our *in vitro* and *in vivo* findings indicated that the PI3K/Akt signaling pathway serves as a critical mediator of LAMC1's regulatory effects on the EMT process. Actually, existing evidences indicated that LAMC1 promotes the Warburg effect in hepatocellular carcinoma cells by regulating PKM2 expression through the AKT pathway [36]. Similarly, Wang et al. discovered that LncRNA SNHG6 promotes EMT via LAMC1/PI3K/Akt cascades [18]. Moreover, the PI3K/AKT signaling pathway has been substantiated to be intricately involved in the modulation of EMT within RPE cells in the context of DR [14]. For example, hyperglycemia-induced oxidative stress, inflammation, and other metabolic alterations in DR contribute to the dysregulation of the PI3K/AKT signaling pathway, which ultimately lead to endothelial dysfunction, aberrant neovascularization, and retinal tissue damage [37], [38]. These findings demonstrated that LAMC1 facilitates high glucose-induced EMT through the activation of the PI3K/AKT signaling pathway, thereby exacerbating the progression of the disease.

While our study has embarked on a comprehensive exploration of DR development mechanisms and immune response changes, certain limitations persist. The reliance on samples from public databases curtails our access to additional clinical data for further stratified analyses based on clinical characteristics. Furthermore, we only conducted in-depth research on LAMC1, and other genes may also play a role in regulating the function of retinal epithelium, which is worth further investigation. Moreover, in our investigation of LAMC1, the findings were solely validated through *in vitro* experiments and rodent models, which may not fully replicate human physiological conditions, necessitating further clinical evidence for comprehensive validation. Finally, we have noticed that these differentiated genes may be related to stromal regulation, which also deserves further investigation.

## Conclusion

In summary, we identified LAMC1 as a critical regulator of EMT in DR for the first time. Moreover, we systematically elucidated that LAMC1 influenced EMT progression through PI3K/AKT axis, revealing a mechanistic link between extracellular matrix components and cellular plasticity. Thus, this study offers the opportunity for treating DR by targeting LAMC1/PI3K/Akt axis.

## CRediT authorship contribution statement

**Shiqi Yao:** Writing – review & editing, Visualization, Project administration, Formal analysis. **Yanlin Gao:** Writing – review & editing, Writing – original draft, Visualization, Project administration, Data curation, Conceptualization. **Lei Liu:** Writing – review & editing, Writing – original draft, Supervision, Conceptualization.

## Ethics approval and consent to participate

All animal-related experimental procedures were approved by the Animal Ethics Committee of Tianjin Eye Hospital (2025-SYDWLL-000401).

## Consent for publication

Not Applicable

## Funding

Sponsored by Tianjin Health Research Project (GrantNo.TJWJ2025MS037); Fund program: Tianjin Key Medical Discipline Construction (TJYXZDXK-3-004A-3)

## Code availability

Not applicable

## Declaration of Competing Interest

The authors declare that they have no known competing financial interests or personal relationships that could have appeared to influence the work reported in this paper.

## Data Availability

The datasets used or analyzed during the current study are available from the corresponding author on reasonable request.

## Footnotes

Appendix A Supplementary data associated with this article can be found in the online version at doi:10.1016/j.jphyss.2025.100045.
