# Supplementary material for: LAMC1 aggravates diabetic retinopathy through PI3K/AKT signaling-regulated epithelial-mesenchymal transition in retinal pigment epithelial cells
Source: J Physiol Sci. 2025 Oct 1;75(3):100045. doi: 10.1016/j.jphyss.2025.100045 (PMC12550245; doi:10.1016/j.jphyss.2025.100045)
Supplement: Supplementary file 1 — Supplementary material [file mmc1.docx]

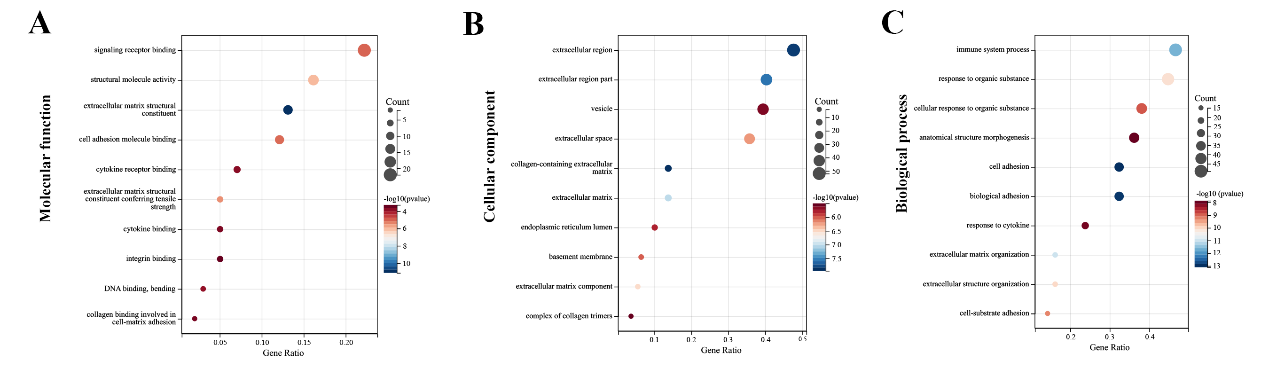


**Supplementary Figure 1. Functional Enrichment Analysis of Module Genes**

A: Molecular function enrichment analysis of module genes; B: Cellular component enrichment analysis of module genes; C: Biological process enrichment analysis of module genes.


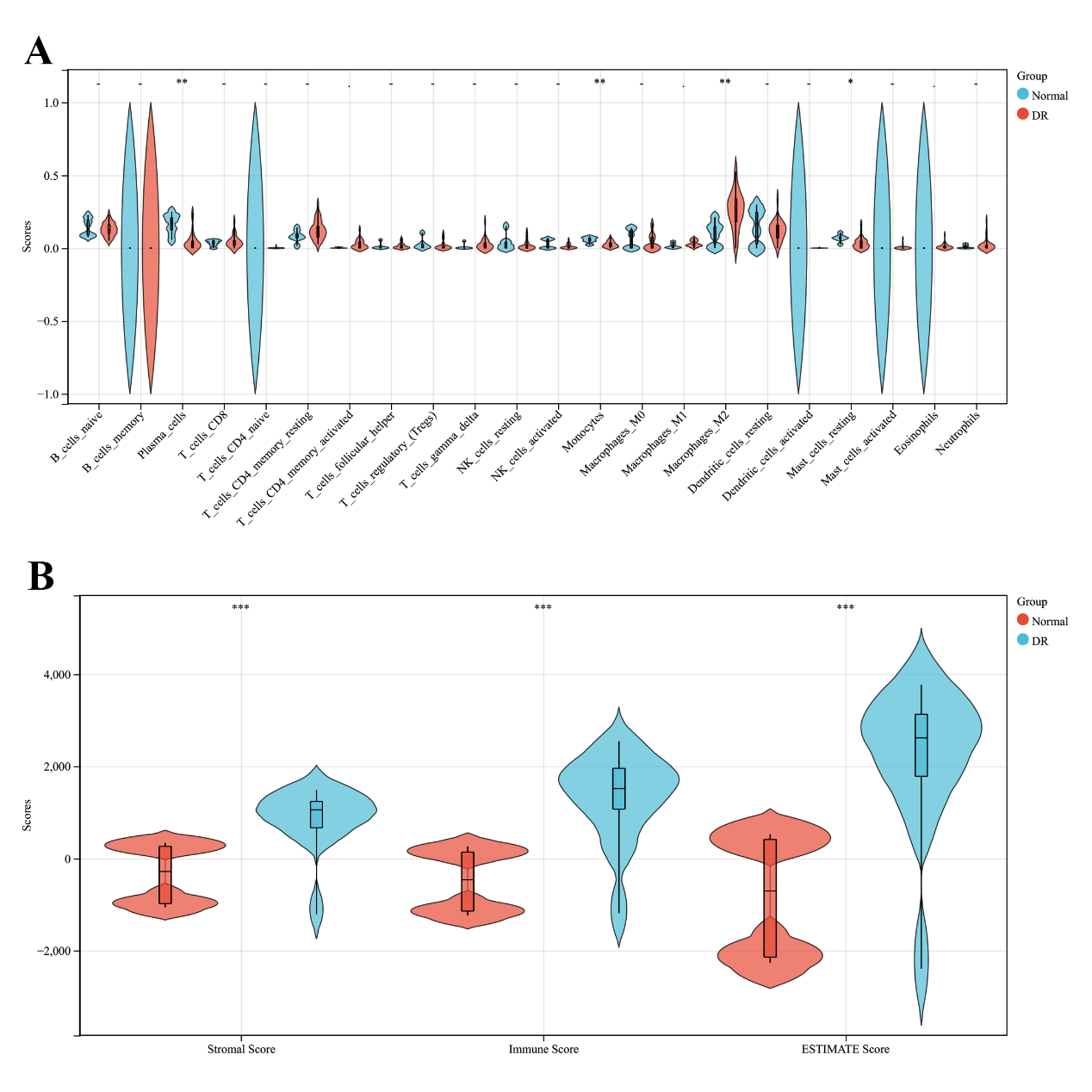


**Supplementary Figure 2: Immune Infiltration Analysis and Association with Diabetic Retinopathy**

A: CIBERSORT algorithm-calculated infiltration scores for 22 immune cell types in DR and normal groups; B: Immune and stromal scores calculated by the ESTIMATE algorithm for DR and normal groups. **P*<.05, ***P*<.01, ****P*<.001.

**
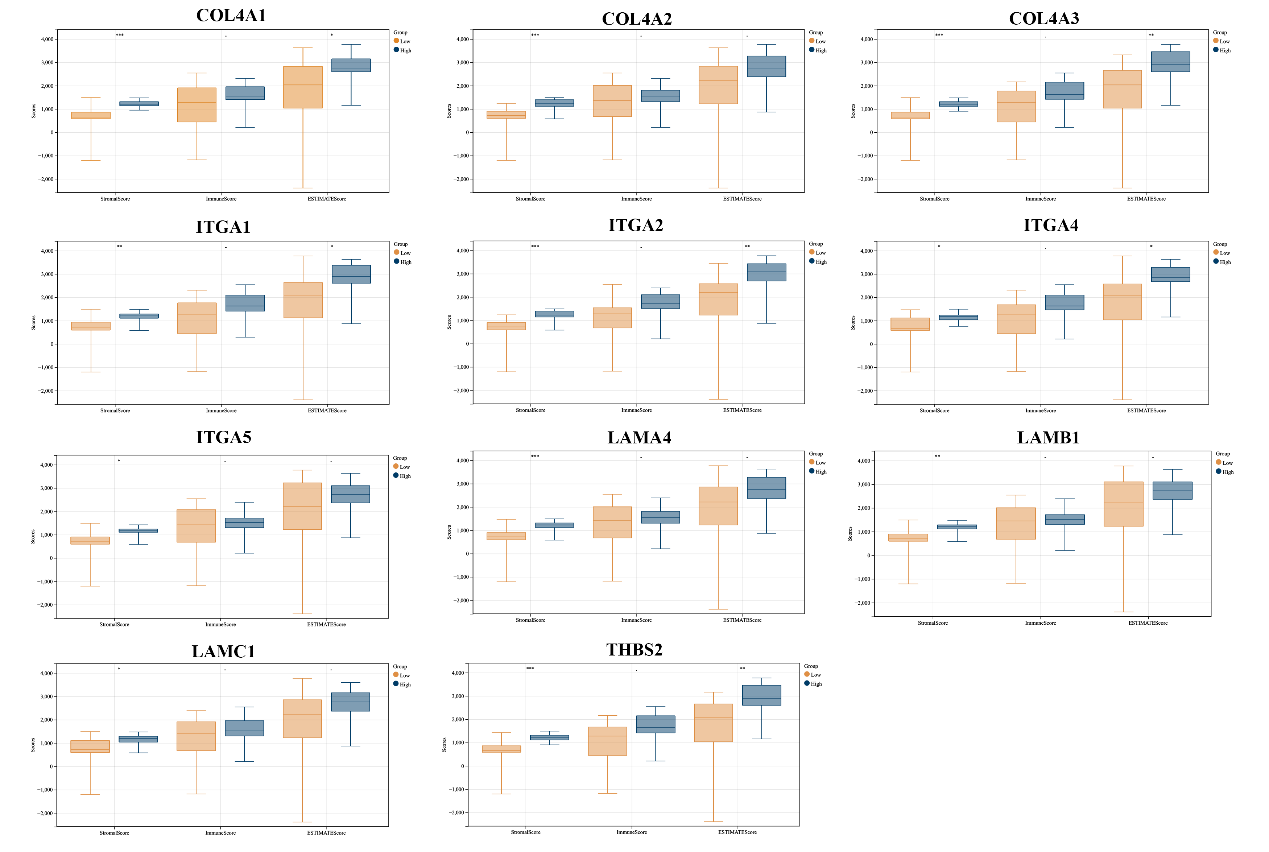
**

**Supplementary Figure 3: Correlation Between Hub Genes and Immune Scores**

Correlation Between Hub Genes and Immune Scores. *P<.05, **P<.01, ***P<.001.
